# Determinants of sexual function among survivors of gynaecological cancers in a tertiary hospital: a cross-sectional study

**DOI:** 10.3332/ecancer.2022.1384

**Published:** 2022-05-05

**Authors:** Maximillar Obora, Lister Onsongo, James O Ogutu

**Affiliations:** 1 Department of Community and Reproductive Health Nursing, School of Nursing Sciences, Kenyatta University, 43844-00100 Nairobi, Kenya; 2 Department of Medical Microbiology and Parasitology, School of Medicine, Kenyatta University, 43844-00100 Nairobi, Kenya; a https://orcid.org/0000-0001-2345-6789

**Keywords:** gynaecological cancer, treatment, sexuality, sexual function, sexual dysfunction, quality of life

## Abstract

**Background:**

Gynaecological cancer impacts approximately three million women globally. The problem is much more intense in resource-limited countries. Sexual health is a critical aspect of gynaecological cancer treatment and an important component of quality of life (QoL).

**Aims:**

This study aimed to assess the determinants of sexual function among survivors of gynaecological cancer.

**Method:**

This was a cross-sectional study. The simple random sampling technique was used to recruit survivors of gynaecological cancers aged 18 years and above on follow-up in a tertiary hospital in Kenya.

**Tools:**

The study used the socio-demographic survey, Body Image Scale, Multidimensional Perceived Social Support Scale and Female Sexual Function Index.

**Results:**

Cervical cancer was the most common gynaecological malignancy among respondents (51%). The mean total score of the Female Sexual Function Index was significantly low at 10.0 (cut off = 26.5). The majority (85%) of respondents had sexual dysfunction. The most commonly affected sexual domain was lubrication at a mean value of 0.91 (SD = 1.58). Age (aOR = 0.05, 95% CI: 0.003–0.16, *p* = 0.005), cancer stage 3 (aOR = 9.81, 95% CI: 1.34–20.56, *p* = 0.035) and social support (aOR = 1.29, 95% CI: 1.05–1.59, *p* = 0.015) were independent predictors of sexual dysfunction.

**Conclusion:**

The prevalence of sexual dysfunction among gynaecological cancer survivors remains significantly high. Having cervical cancer was the most significant predictor of sexual dysfunction in this study population.

**Recommendation:**

There is a need for further studies to improve the sexual life and hence the QoL among survivors of gynaecological malignancies.

## Introduction

Cancer is the most significant worldwide pathological health problem with wide geographical variation in incidence, and it has additionally become an important item in each country’s health agenda [1]. Gynaecological malignancies are major contributors to morbidity and mortality in women globally. It is estimated that more than 1.39 million women are living with gynaecological malignancies worldwide [2].

The burden of gynaecological cancer in developing countries appears huge. In these countries, gynaecological cancers account for 25% of all new cancers diagnosed among women aged up to 65 years compared to 16% in the developed world. According to a recent report, developing countries accounted for 820,265 cases [3].

All women are at risk of gynaecologic cancer. Gynaecological cancers represent the second most common malignancies affecting women in Kenya [4]. On the contrary, significant advances in the treatment of gynaecological cancers have been made [5–7]. There is no doubt that gynaecological cancer is а stressful experience by creating heavy psychological trauma for the woman and has а great impact on the psychological and emotional health, sexual issues, body image and quality of life (QoL) of women [8–12].

Addressing survivorship and QoL issues remains a challenge [9, 10, 13]. Survival for gynaecological malignancies is upwards of 90% for most early-stage uterine and cervical cancers and has also increased among those with aggressive primary cancers [14–17]. Cancer survivorship is associated with distressing long-lasting adverse effects that negatively impact women’s sexual health [1, 9–11, 15, 18].

Gynaecological cancer and its treatments can affect one or more phases of the sexual response cycle through alterations of sexual function. The high curability of cervical cancer, when detected early, combined with the latest scientific advances in medical treatment, has contributed to the greater survival of patients. However, treatment of this neoplasm can, on the other hand, lead to late adverse effects, primarily related to radiotherapy, caused by its action on healthy tissue and organs adjacent to the tumour [10, 17, 19].

Approximately 50% of gynaecological cancer survivors present with sexual dysfunction [20–24]. Most healthcare professionals fail to routinely assess sexual function needs, despite the high prevalence of sexual dysfunction, which negatively impacts the QoL and sexual function [25–32]. Studies examining cancer survivors with sexual dysfunction have mainly concentrated on women with breast cancer [33–37]. There is limited literature regarding sexual health or dysfunction among a diverse group of women with gynaecological malignancies. Studies have recognised the need to point out sub-groups of gynaecological cancer survivors at risk for sexual dysfunction [10, 17, 19, 38].

Sexual dysfunction is one of the most distressful symptoms among cervical cancer survivors. Cancer treatment, including radiotherapy, results in a high degree of vaginal morbidity and persistent sexual dysfunction. The vaginal symptoms reported after cervical cancer treatment include sore membranes, reduced lubrication and genital swelling, which severely affect women’s sexual health [11, 21, 27].

The illness process and various treatment modalities negatively affect the emotional, psychological, physiological, body image, QoL and sociocultural well-being, severely affecting the affected women and their spouses [8, 15, 17, 39–41]. Physiological consequences of gynaecological cancers and treatment, including radiotherapy, result in a high degree of vaginal morbidity, persistent sexual dysfunction, decreased lubrication, vaginal dryness, atrophy, dyspareunia and reduced libido [11, 17, 21, 27]. The vaginal symptoms reported after cervical cancer treatment include sore membranes, reduced lubrication and genital swelling, which severely affect the women’s sexual health [10, 22, 24]. Others are loss of genital sensation, post-coital bleeding, premature menopause, inhibited orgasm, inhibited desire and inhibited arousal [42–44]. With regard to psychological components, some patients may experience sadness, guilt, anger, depression, body image changes, fear of cancer recurrence and a negative sexual self-schema [8, 13, 15, 25, 39]. The social consequences of gynaecological cancer diagnosis and treatment manifest in relationships with friends, family and the significant other. The emotional responses either by accepting or rejecting the gynaecological cancer diagnosis and treatment regimen may affect the patient’s relationship, psychological well-being and partner [8, 45]. Changes in an intimate relationship after cancer diagnosis and treatment can lead to a strenuous relationship, difficulties in forming a new relationship, re-prioritisation of sex and bargaining of sexual intercourse [46–48].

Despite substantial studies recording the detrimental outcome of malignancies on sexual functioning and gratification, there is a paucity of studies on successful management programmes on sexual dysfunction within oncology care settings [7, 23, 49]. The magnitude of sexual dysfunction remains largely underdiagnosed, and often goes unaddressed and under-treated due to various challenges regarding the discussion of sexual problems in public, and is often dismissed as a normal side effect of cancer treatment [11, 21, 27].

Majority of the patients who experience sexual difficulties rarely discuss problems freely with their healthcare providers for the reason that sex is an embarrassing topic to discuss in public and is considered a taboo in many parts of the world, especially in the African and Asian communities [42, 50, 51]. Sexual/reproductive health education is rarely carried out or limited in specific communities, resulting in misconceptions, uncertainty, panic and worries about sexual activity [32, 50].

Discussion of sexuality after gynaecological cancer treatment is often tackled from a biomedical point of view, with the centre of attention being the physiological aspect of sexual functioning [1, 3, 33]. Additionally, earlier studies on malignancy and sexuality concentrated on sexual performance or assessing characteristics that predicted sexual dysfunction. Most studies focused on physical, socio-demographic factors, type of management, relationship context or other attributes related to reduced sexual function post-cancer. However, limited focus has been on the psychological and social effects of sexual function. Additionally, many studies on the impact of cancer on psychosexuality are carried out in western cultural settings [52]. This has resulted in calls for action into conducting more studies on cancer and sexuality by embracing an integrative point of view and examining physical, psychological, social and relational aspects of sexual change in racially diverse patients [18, 47].

## Method

### Objectives

The study was carried out to assess the determinants of sexual function among survivors of gynaecological cancers in a tertiary teaching and referral hospital.

Specific objectives were (1) to assess the association between clinical characteristics, socio-demographic characteristics and sexual function among survivors of gynaecological cancers; (2) to assess the relationship between psychosocial determinants and sexual function among survivors of gynaecological cancers; and (3) to determine the predictors of sexual function among survivors of gynaecological cancers.

### Study design and setting

A cross-sectional study design was utilised. The study was conducted in a tertiary hospital in Kenya. The hospital is the largest public referral hospital that offers comprehensive cancer care services at subsidised rates. It has an inpatient bed capacity of over 2,000 and provides outpatient services to approximately 27,000 cancer patients annually. Data were collected from the period of March 2021 to April 2021.

### Sample

The sample size was determined using Fischer *et al* [69] formula, where *z* = 1.96, *p* = 0.5 and *d* = 0.05. The sample size obtained was corrected considering a target population of 150 female patients attending an outpatient clinic, aged 18 years and above with confirmed diagnosis of gynaecological cancers, on treatment and regular follow-up in a tertiary hospital – Cancer Treatment Centre. A total of 108 participants were recruited in the study.

### Sampling technique

The simple random sampling method was used to recruit participants in the study whereby yes/no papers were written and placed in a box and mixed well, and then drawn out one at a time. Those who picked yes paper were eligible to participate. To ensure that a participant was not recruited more than once, the researcher and research assistants sensitised the participants through a health talk at their respective clinics about the study and sampling process. Once the participant was selected, the participant’s file was marked with a red sticker for easier identification on the day when they attended their routine clinics, and for those who had participated and were not selected, their files were marked with a yellow sticker, and they were not involved in sampling process again. Simple random sampling was repeated daily from Monday to Friday for 1 month. On average, 5 participants were enrolled per day until the targeted sample size of 108 was reached.

### Inclusion criteria

Women were recruited to participate in the study if they were currently on treatment or had undergone therapy within the previous 3 years. To have a more homogeneous sample, the inclusion criteria were (i) adult female patients aged 18 years and above; (ii) currently in a relationship with a spouse or long-term sexual partner; (iii) with a history of having actively engaged in sexual activity over 1 month prior gynaecological cancers diagnosis and treatment; and (iv) able to read, write and speak English or Kiswahili. Women with the following conditions that potentially would influence their sexual life were excluded: the presence of other malignant diseases, impaired mental function, previous history of sexual dysfunction, history of female genital organ surgery before the diagnosis of gynaecological cancer, end-stage renal disease or a dependent functional status.

### Recruitment

After establishing a rapport with the patients, the first author approached eligible women at an outpatient clinic of a tertiary hospital – Cancer Treatment Centre. The aims and procedures of the study were explained. Written informed consent was obtained from the patients who agreed to participate in the study. An interviewer-administered questionnaire was used to collect data from participants who expressed difficulty in reading questionnaires but were willing to respond.

### Data collection tools

Demographic data, such as age, marital status, level of education and employment status, were collected with a survey questionnaire. Clinical information, such as cancer staging and type of gynaecological cancer, treatment modalities and treatment duration, were extracted from medical records. The instrument used to evaluate the sexual function, body image and perceived social support for patients with gynaecological cancer survivors was collected with self-report instruments described below.

A 19-item Female Sexual Function Index was developed by Rosen *et al* [53] to measure the orgasm, arousal, lubrication, desire, pain and satisfaction domains of female sexual function over the past 4 weeks. Questions were scored based on a 0 or 1–5 scoring system represented on an ordinal Likert scale. The total scores ranged from 2 to 36, where the clinical cut-off scores of 26.55 and below indicated sexual dysfunction. The validity and reliability of the tools were verified. Internal consistency of the tool was assessed using Cronbach’s alpha. Six subscales of the Female Sexual Function Index had excellent internal reliability (Cronbach’s alpha > 0.97 for all subscales) and good test–retest reliability scores ranged from 0.79 to 0.88.

A 10-item Body Image Scale was developed by Hopwood *et al* [54], collaborating with the European Organisation for Research and Treatment of Cancer Quality of Life Study Group, to measure body image changes in patients with cancer. Items are rated on a 4-point scale ‘not at al’ (score 0), ‘a little’ (score 1), ‘quite a bit’ (score 2) and ‘very much’ (score 3). The total scores ranged from 0 to 30. Zero scores indicated no symptoms/distress, and higher scores indicated increasing symptoms/distress. A suggested body image score of ≥10 was the clinical cut-off point for body image distress. Internal consistency was measured using Cronbach’s alpha reliability coefficient, with a minimum value of 0.70 for retaining items.

A 12-item Multidimensional Scale of Perceived Social Support was developed by Zimet *et al* [79] to measures social support from three sources: family, friends and significant other; social support was rated on a 7-point Likert scale, with responses ranging from 1 = very strongly disagree, 2 = strongly disagree, 3 = mildly disagree, 4 = neutral, 5 = mildly agree, 6 = strongly agree to 7 = very strongly agree. The total response scale ranged from 12 to 84, of which scores ranging from 12 to 35 indicate low support; 36–60 indicate medium support; and 61–84 indicate high support. An approach by the mean scale ranging from 1 to 2.9 could be considered low support; 3–5 could be considered moderate support; and 5.1–7 could be regarded as high support. Internal consistency of the scale was good, with a Cronbach’s alpha of 0.91.

### Data analysis

Data were analysed using Statistical Package for the Social Sciences (SPSS) version 25. Descriptive statistics, such as mean, standard deviation, frequencies and percentages, were used to summarise study variables. Chi-squared tests were used to test the association between socio-demographic, clinical characteristics and FSFI. Pearson’s correlation test was used to investigate the relationship between psychological, social and sexual functions. A binary logistic regression model was used to identify the predictors of sexual function. Significance was accepted at *p* < 0.05.

### Ethical considerations

The university’s Ethics Review Committee, the National Commission for Science, Technology, Innovation, and the Hospital Administration approved the study. The informed consent form was signed before data collection. Confidentiality and privacy were assured throughout the study by maintaining the anonymity of the participants and storing the data in password-protected files.

## Results

### Socio-demographic and clinical characteristics

The average age of the respondents was 53.4 (SD ± 12) years; 58.3% (*n* = 63) of the respondents had secondary education as their highest level of education; 77.8% (*n* = 81) were unemployed; and 69.4% (*n* = 75) were married. The majority of respondents (50.9%,* n* =55) had cancer of the cervix, and 55.6% (*n* =60) had stage 3 cancer. Chemotherapy (48.1%, n =52) was the commonest mode of treatment (Table 1).

### Psychological and social factors

The average score of patients who were dissatisfied with the changes that had occurred to their body due to the disease and its related treatment was *M* = 1.66 and SD = 0.96. The highest rating was feeling self-conscious about their appearance with an average score of *M* = 2.06 and SD = 1.0. Family support was the most significant to the patients with an average score of *M* = 3.44 and SD* =* 1.16 (Table 2)

### Sexual function

The majority of respondents (85%, *n* = 92) had sexual dysfunction. Over half of the respondents (55.6%, *n* = 60) had their physicians discuss sexual functioning issues. The majority of respondents (69%, *n* = 75) were not referred to a sexual specialist counsellor or social support after treatment and 55.6% (*n* = 60) had their physicians discuss sexual functioning issues. Lubrication was the most prevalent problem (*M* = 0.91, SD = 1.58) (Table 3).

### Correlation between socio-demographic, clinical characteristics, cultural factors and psychosocial and sexual function among survivors of gynaecological cancer

Age of the patient (*p* = 0.001), patient’s level of education (*p* = 0.002), employment status (*p* < 0.0001), lifestyle adaptation activities (*p* = 0.047), type of cancer (*p* = 0.015) and cancer staging (*p* = 0.024) were significantly associated with sexual function. Type of cancer (*p* = 0.015) and cancer staging (*p* =0.024) were significantly associated with sexual function. A significant positive relationship existed between body image and sexual function (*r* = 0.240, *p* = 0.013). There was a significant negative relationship between social support and sexual function (*r* = 0.127, *p* < 0.0001) (Table 4).

### Predictors of sexual function among female patients with gynaecological cancers

The findings revealed that the respondents’ age, employment status, type of gynaecological cancers and cancer staging were significant predictors of sexual dysfunction. Patients aged less than 50 years were 0.14 times less unlikely to suffer from sexual dysfunction than those aged 50 years and above (*p* = 0.004, OR = 0.142, 95% CI = 0.038, 0.533). In assessing employment status, employed participants were 0.3 times less likely to have sexual dysfunction (*p* = 0.002, OR = 0.321, 95% CI = 0.002, 0.641). At the same time, self-employed people were 0.1 times less likely to have sexual dysfunction (*p* < 0.001, OR = 0.095, 95% CI = 0.027, 0.339) compared to unemployed respondents. Respondents who had cancer of the cervix were seven times more likely to have sexual dysfunction (*p* = 0.016, OR = 7.833, 95% CI = 1.469, 13.150), while patients who had cancer of endometrium were five times more likely to have sexual dysfunction (*p* = 0.018, OR = 5.120, 95% CI = 1.498, 10.450). Respondents with stage 4 cancer were five times more likely to have sexual dysfunction (*p* = 0.008, OR = 5.211, 95% CI = 1.552, 17.495).

The findings revealed that a decrease in the social support scale scores among the respondents had a 10.2% increased chance of having sexual dysfunction among the respondents (*p* = 0.037, OR = 1.102, 95% CI = 1.006, 1.207) (Table 5).

### Independent predictors of sexual function among female patients with gynaecological cancers

Multivariable analysis conducted, as shown in Table 6, revealed that the age of the patient, cancer staging and social support were independent predictors of sexual dysfunction. Patients who were aged less than 50 years were 95% less likely to have sexual dysfunction compared to those aged ≥50 years (aOR = 0.05, 95% CI: 0.003–0.16, *p* = 0.005). Patients who had stage 3 cancer were ten times more likely to have sexual dysfunction compared to those with stage 1 cancer (aOR = 9.81, 95% CI:1.34–20.56, *p* = 0.035). A decrease in the social support score was associated with a 29% increase in the likelihood of sexual dysfunction among patients with gynaecological cancers (aOR = 1.29, 95% CI: 1.05–1.59, *p* =0.015).

### Discussion

To the best of our knowledge, this is the first comprehensive quantitative study to address specific sexual functioning and activities among survivors of gynaecological cancer in Kenya. The present study found that the mean total FSFI score was 10.0. This study revealed the worse sexual functions in all domains. The present study found that the majority of respondents (85%) reported sexual dysfunction. Furthermore, most of the respondents (69%) were not referred to a sexual specialist counsellor or social support after treatment. Cervical cancer (51%) was the commonest among respondents, and 55% were stage 3. Poor sexual activity was linked with older age, type of gynaecological cancers, cancer stage, employment status and level of education. Additionally, sexual dysfunction is indicated by its substantial positive association with a higher level of psychological distress due to body image alteration and negatively correlates with decreased social support.

The study’s findings showed that the mean Female Sexual Function Index score was 10 among patients with gynaecological cancer. These findings are comparable to Rai [55], in Nepal, who found that the total FSFI score was 8.1 among cervical cancer survivors. However, the mean FSFI score from the present study was lower than Lara and Maree’s [56] study in South Africa, where the FSFI average score was 15.1. Our present study found that 85% of the respondents reported sexual dysfunction, which is much higher compared with previous studies by Pinar *et al* [57] and Liu *et al* [58], who indicated that 80% of Turkish and 82% of Chinese women with gynaecological cancers had sexual dysfunction. This may be attributed to advance cancer stages and the negative consequences of treatment modalities. The majority of respondents were symptomatic, in cancer stages III and IV and undergoing chemotherapy and radiotherapy. Chemo-radiation is the treatment of choice, especially in advanced cancer stages and palliative care. However, it is associated with higher levels of sexual dysfunction [59, 60]. Another reason is that gynaecological malignancies and their treatment affect women physically and psychosocially. Sexual function is among the most affected areas because the cancer is located around the genital organs [10, 13, 22, 57]. In addition, the study findings revealed that over half of the physicians did not prepare or screen sexual function issues with their patients’ pre- and post-treatment; some referred those who had sexual problems to sex specialists for further evaluation, which could be an essential factor contributing to the higher prevalence of sexual dysfunction. On the other hand, most women cherish talks of their sexual activity with a known healthcare provider instead of being referred to someone else, and some reject counselling or referral to a sexual counsellor.

This present study found no significant differences in the distribution of the FSFI domain scores. However, lubrication was the most commonly affected domain. Similar to our study’s findings, Correia *et al* [61] found that patients who lacked lubrication were at a high rate for sexual dyspareunia among cervical cancer survivors. Contrary to our study findings, several studies indicated that arousal and pain were the most affected domains – for instance, studies by Zhou *et al* [62] and Rai [55]. Decreased lubrication may be due to adverse effects of treatment modalities such as chemotherapy, pelvic radiation and removal of ovaries coupled together with physiological changes or decline in biological function, primarily decreased oestrogen levels [43, 61, 63]. In the current study, most of the respondents were older, and a significant number were undergoing chemotherapy.

The study’s findings revealed that the age of the patient was statistically significantly associated with sexual function. This is consistent with the findings in Kaviani *et al* [64] and Rai’s [55] studies, who indicated that age was associated with sexual dysfunction. These findings were in disagreement with Guler [41] and Golbasi and Erenel [65], who reported that age never influences the sexual quality of life (SQoL). One explanation is that in our study sample majority were older women with poor lifestyle adaptation, unemployed and lower level of education. One may conclude that older women experience more problems with sexual function. This could be explained by physiological and physical changes during and after menopause, such as decrease in oestrogen levels. Additionally, health problems, such as cardiovascular disease, hypertension and diabetes, may contribute to sexual dysfunction as women age. Likewise, the treatment modality for the gynaecological malignancies in older patients is usually planned, keenly selected and sometimes modified [66].

This study also found an association between the level of education and sexual function, which agrees with previous reports by Rai [55]. Of the sexual dysfunctional women, the majority had a lower level of education. Another study by Kaviani *et al* [64] concluded that a significant positive correlation existed between sexual knowledge and sexual attitude. Contrary to Sekse *et al*’s [7] study, Golbasi and Erenel [65] and Guler [41] found no association between education and sexual function. This probably confirms that as the couple’s knowledge, adequate education and awareness of sexual issues increases, their attitude becomes more positive [62]. Educated women can be a good source of information transfer to family and society. Additionally, education creates awareness of the disease symptoms and their effects which is a fundamental requirement to help-seeking behaviours [67]. Insufficient information and inadequate knowledge about the sexual function can result in a higher level of misconception and hesitancy [42].

This study further revealed that employment status was statistically significantly associated with sexual function. These results are similar to studies conducted by Sekse *et al* [7] and Zhou *et al* [62], who found that among sexually inactive women, majority (48%) were retired and 38% were employed. In our study, most of the respondents who were sexually dysfunctional were unemployed. One possible reason is that disease burden and a poor health condition could result in psychological distress and social isolation due to lack of employment, job loss and workplace discrimination [67]. On the contrary, Pinar *et al* [57] revealed that employment status did not affect sexual function.

Our study found an association between lifestyle adaptation and sexual function, of which a good number of the respondent did not engage in any lifestyle adaptation activities. These research findings are consistent with Bifulco *et al* [68], who reported inadequate physical activities among gynaecological cancer survivors. They further stated that younger patients showed improved an lifestyle, apart from smoking behaviours, but they added on more weight than older patients. On the contrary, Sekse *et al* [7] and Rai [55] indicated that regular physical activity and smoking were not associated with sexual function. This may be attributed to a poor lifestyle adaptation. Lifestyle adaptation is one of the recommended sexual rehabilitative strategies [6].

The study demonstrated that the type of gynaecological cancers and cancer staging were associated with sexual function. This is similar to studies carried out by Correia *et al* [61], Fischer *et al* [69] and Shankar et al. [23], who found that cervical cancer and ovarian cancer survivors in advanced cancer stages were more associated with sexual dysfunction. One possible explanation is that over half of the respondents were in advanced cancer stage III in our study. The advanced cancer stage is associated with the worst sexual activity. In most cases, a combination of different treatment modalities, such as radiotherapy and chemotherapy used in managing metastatic diseases, often have a profound negative effect on women’s sexual function [6, 16]. Surprisingly, this study found no statistically significant association between treatment modalities and sexual function. Another possible explanation is that gynaecological cancer affects sexual/reproductive organs; lack of accessibility to the services, financial burden and lack of screening tools acts as barriers in accessing medicine. This may force patients to seek treatment when symptomatic and in advanced stages with poor performance status [70]. The study’s findings are in disagreement with those of Sekse *et al* [7].

The present study found a positive statistically significant relationship between the psychological domain (body image changes) and sexual function. These findings are comparable to studies showing that the body’s image changes during cancer treatment, often resulting in sexual dysfunction, and patients may or may not recover gradually even after 5 years of treatment [69, 71]. These results may arise because the present study found considerably higher body image scores among respondents, especially those who were dissatisfied with their scar, self-conscious about their appearance, felt less physically attractive and those who were dissatisfied when dressed, which signifies that psychological distress/dissatisfaction has a negative impact on sexual function. Similar findings were echoed in the studies by Izycki *et al* [72], Li *et al* [51] and Ussher *et al* [47], who found that combining various treatment modalities promoted the manifestation of both adverse effects on psychological well-being and sexual function. Ussher *et al* [47] indicated that previous studies on psychological and relational aspects of variations to sexuality post-treatment majorly used quantitative study design, which prevents an evaluation of the subjective experience and meaning of such alterations for cancer patients. Moreover, some qualitative studies have evaluated sexual changes post-cancer and how sociocultural discourses shape the experience and understanding of sexuality by differentiating between normal and abnormal sexual activity. However, this study has relied on a few participants, majorly with reproductive cancers, limiting insights into individuals’ experience with other cancers. Mixed-method designs with larger samples of data are recommended.

This study showed a positive statistically significant relationship between social support and sexual function. This study found low social support scores among respondents compared with published normative data, which indicated low social support. These findings agree with Golbasi and Erenel [65], who found that social support correlated with sexual function, of which patients with low social support presented more with sexual dysfunction. They concluded that women with gynaecological cancers’ SQoL have moderate and social support, primarily support from friends, family and the significant others, and may positively affect the SQoL. A healthy relationship with the spouse is valuable and one of the recommended sexual rehabilitative strategies which should be encouraged because it acts as a supporting factor in the coping process [4, 21]. This is also similarly seen in studies by Pfaendler *et al* [73] and Izycki *et al* [72], who indicated that gynaecological cancer survivors with a low social support system and poor coping mechanism were more likely to manifest with poor QoL and sexual problems. On the contrary, Fischer *et al* [69] found higher social support among ovarian cancer survivors.

From the regression analysis, our study’s findings revealed that the respondent’s age, employment status, type of gynaecological and cancer staging were significant predictors of sexual function among the respondents. This study found that respondents aged less than 50 years were less unlikely to suffer from sexual dysfunction than those aged 50 years and above. These findings are in line with Sekse *et al* [7] and Pinar *et al* [57], who stated that women who were 53 years and below were sexually active compared to those who were 61 years and above. It can be argued that sexual activity decreases with an increase in age. Older women are less likely to undertake regular gynaecological screening tests than younger women, which often results in late cancer detections [66]. Additionally, most women reported being sexually inactive but are content with the lack of sexual intercourse. One would argue that maybe menopausal women are used to vaginal dryness and other comorbidities before gynaecological cancer diagnosis and treatment [7, 74]. Older adults who are sexually active often face barriers to proper care from healthcare providers who view their sexuality as forbidden. They suspect that the majority of people assume that they are asexual; hence, they tend to feel sexually invisible. Older adults who internalise these beliefs are often ashamed of their bodies and are unlikely to express their sexual desires for fear of judgment and exclusion [47].

Kew *et al* [75] reported that intimate relationships in later life are culturally taboo. However, many older women remain sexually active beyond 70 years of age. On the contrary, Bifulco *et al* [68], Boa and Grénman [6] and Pfaendler *et al* [73] found that younger women were significantly more affected by sexual dysfunction than older women.

This study revealed that unemployed respondents were more likely to suffer from sexual dysfunction than employed and self-employed women. These findings were consistent with Najjar *et al* [76], Pinar *et al* [57] and Zhou *et al* [62], who found that the increased rate of unemployment, especially among patients with the lowest level of education, was positively associated with the presence of poor sexual activity.

This study revealed that respondents who had cancer of the cervix were more likely to have sexual dysfunction compared to respondents with other gynaecological cancers. Furthermore, our study demonstrated that respondents who had stage 4 cancer were more likely to have sexual dysfunction than respondents in stages I, II and III. This research is the first study in our country to indicate that cervical cancer survivors manifest more with sexual dysfunction compared with other gynaecological malignancies. One possible explanation is that over 50% of our study sample consisted of respondents with cervical cancer, probably in the advanced tumour staging. Additionally, Muliira *et al* [70] and Boa and Grénman [6] reported that cervical cancer ranks second after breast cancer, common in middle- and low-income countries affecting women at an earlier age/reproductive stages. It is easily detected due to the availability of screening tools compared with other gynaecological malignancies diagnosed in late stages.

Furthermore, Williams *et al* [80] revealed that cervical cancer survivors delayed seeking medical attention compared with endometrial cancer survivors because the bleeding was less likely to be considered a symptom of disease in premenopausal women with cervical cancer. Interestingly, most research studies concentrated on cervical malignancy compared with other gynaecological malignancies [59]. However, our study’s findings are in disagreement with several previous studies, for instance, Sekse *et al* [7], Frimer *et al* [5] and Pinar *et al* [57], which reported that endometrial cancer patients manifested more with sexual dysfunction compared with other gynaecological cancers. Sekse *et al* [7] argued that ovarian, cervical, endometrial and vulvar cancers adversely affect sexual activity. However, there are a small number of studies that describe and compare sexual performances and functioning as per the diagnosis.

The study found that the respondents who had decreased social support had an increased chance of sexual dysfunction. These findings mirror the findings from studies Izycki *et al* [72] and Donkers *et al* [77], who found that a lower social support system was linked with poor QoL and sexual problems among survivors of gynaecological cancer. One possible reason is that respondents have low-to-moderate social support from friends, family and the significant other. On the other hand, women with low social support are vulnerable to comorbidity diseases and risky health behaviours, such as smoking and mental problems that result in poor QoL and sexual issues [78, 79]. Pfaendler *et al* [73] suggest that providing supportive care is essential to coping with a cancer diagnosis, treatment and emotional consequences throughout survivorship, reducing the magnitude of sexual dysfunction and improving the QoL.

## Limitations

This research study was quantitative by design, of which the majority of participants were survivors of cervical cancer, limiting insights into subjective and individuals’ experience with other gynaecological cancers. Mixed-method designs with a larger sample size comprising an equal number of survivors of different types of gynaecological cancers are recommended in future studies. In addition, the cross-sectional design of this study may not allow us to infer a causal relationship between treatment and sexual dysfunction as we could not objectively assess the pre-treatment sexual function in the participant.

## Conclusion

The FSFI score among respondents with gynaecological cancer was low, with an 85% prevalence of sexual dysfunction. Based on the study’s findings, the age of the patient, patient-level of education, employment status and lifestyle adaptation activities were socio-demographic factors associated with sexual function among respondents. The type of cancer and cancer staging were clinical characteristics associated with sexual function. Psychological (body image) and social factors (social support) were related to sexual function. Age, cancer stage 3 and social factors were independent predictors of sexual dysfunction.

## Author contributions

Conceptualisation: M.O., with L.O. and J.O.O.’s supervision; Literature search: M.O., with L.O.’s supervision; Study design: M.O., with L.O. and J.O.O.’s supervision; Data collection: M.O., with L.O. and J.O.O.’s supervision; Data analysis: M.O., with L.O. and J.O.O.’s supervision; Manuscript preparation: M.O., with L.O.’s supervision; Writing and editing of manuscript: M.O., with L.O. and J.O.O.’s supervision. All authors read and approved the final version of the manuscript.

## Conflicts of interest

The authors declare that there is no conflict of interest.

## Funding sources/sponsorship

This study was self-funded. There was no external funding.

## Figures and Tables

**Table 1.** Socio-demographic and clinical characteristics of the respondents.

Characteristics	Mean ± SD	(*n*)	(%)
**Age**			
Mean (SD)	53.4 ± 12.2		
**Level of education**			
None		6	5.6
Primary		32	29.6
Secondary		63	58.3
College		6	5.6
Degree		1	0.9
**Employment status**			
Employed		4	3.7
Self-employed		20	18.5
Unemployed		84	77.8
**Marital status**			
Single		10	9.3
Married		75	69.4
Divorced		3	2.8
Separated		4	3.7
Widowed		16	14.8
**Religion**			
Christian		88	81.5
Muslim		20	18.5
**Religious beliefs promote free discussion**			
Yes		108	100
**Lifestyle adaptation activities**			
Drinking		1	0.9
Physical activities		2	1.9
None		105	97.2
**Clinical characteristics**			
**Type of cancer**			
Cancer of the cervix		55	50.9
Cancer of the endometrium		22	20.4
Cancer of the ovary		15	13.9
Cancer of the vulva		12	11.1
Cancer of the vagina		4	3.7
**Cancer staging**			
Stage 1		4	3.7
Stage 2		16	14.8
Stage 3		60	55.6
Stage 4		28	25.9
**Treatment method**			
Surgery		19	17.6
Chemotherapy		52	48.1
Radiation		31	28.7
Hormonal therapy		6	5.6
Duration of treatment			
≤6 months		52	48.1
7–12 months		31	28.7
>12 months		25	23.1

**Table 2.** Psychological and social determinants of respondents’ sexual function.

Body Image Scale: response and perception frequency for individual items	Mean	Std. deviation
Have you been feeling self-conscious about your appearance?	2.06	1.003
Have you felt less physically attractive as a result of your disease or treatment?	1.91	1.072
Have you been dissatisfied with your appearance when dressed?	1.86	0.88
Have you been feeling less feminine/masculine as a result of your disease or treatment?	1.6	0.937
Did you find it difficult to look at yourself naked?	1.81	0.799
Have you been feeling less sexually attractive as a result of your disease or treatment?	1.66	0.959
Did you avoid people because of the way you felt about your appearance?	1.71	0.958
Have you been feeling the treatment has left your body less whole?	1.44	0.752
Have you felt dissatisfied with your body?	1.66	0.822
Have you been dissatisfied with the appearance of your scar?	1.81	0.833
**Average total score**	**17.52**	**7.58**
MSPSS Items		
**Family**		
My family members try to help me	3.29	1.094
I get emotional help and support I need from my family	3.53	1.203
I can talk about my problems with my family	3.46	1.164
My family is willing to help me make decisions	3.49	1.164
**Friends**		
My friends really try to help me	1.74	0.44
I can count on my friends when things go wrong	1.73	0.506
I have friends with whom I can share my joys and sorrows.	1.76	0.49
I can talk about my problems with my friends	1.76	0.43
**Significant Other**		
I have a special person who is a real source of comfort to me	1.78	0.649
There is a special person in my life that cares about my feelings	1.8	0.608
There is a special person who is around when I am in need	1.82	0.609
There is special person with whom I can share my joys and sorrows	1.8	0.623
**Average significant score**	**1.8**	**0.62**
**Average Family score**	**3.44**	**1.16**
**Average Friends score**	**1.75**	**0.47**
**Average MSPSS score**	**29.54**	**6.57**

**Table 3.** Domains of sexual function.

Domain	Questions	Score range	Factor	Minimum score	Maximum score	Score
Desire	1,2	1–5	0.6	1.2	6	2.6
Arousal	3,4,5,6	0–5	0.3	0	6	2.3
Lubrication	7,8,9,10	0–5	0.3	0	6	0.9
Orgasm	11,12,13	0–5	0.4	0	6	1.2
Satisfaction	14,15,16	0 or (1)–5	0.4	0.8	6	1.9
Pain	17,18,19	0–5	0.4	0	6	1.1
Full-Scale Score Range				2	36	10

**Table 4.** Association between socio-demographic and clinical characteristics and sexual function.

	Sexual function		*p*-value
	Normal Sexual function *n* (%)	Sexual Dysfunction *n* (%)	
**Age**			
Less than 50 years	13 (81.3)	35 (38)	0.001
≥ 50 years	3 (18.8)	57 (62)	
**Level of education**			
None	0	6 (6.5)	
Primary	1 (6.3)	31 (33.7)	
Secondary	11 (68.8)	52 (56.5)	0.002
College	3 (18.8)	3 (3.3)	
Degree	1 (6.3)	0	
**Employment status**			
Employed	3 (18.8)	1 (1.1)	
Self-employed	8 (50)	12 (13)	*p* < 0.0001
Unemployed	5 (31.3)	79 (85.9)	
**Marital status**			
Single	1 (6.3)	9 (9.8)	
Married	13 (81.3)	62 (67.4)	
Divorced	1 (6.3)	2 (2.2)	0.558
Separated	0	4 (4.3)	
Widowed	1 (6.3)	15 (16.3)	
**Lifestyle adaptation activities**			
Drinking	1 (6.3)	0	
Physical activities	0	2 (2.2)	0.047
None	15 (93.8)	90 (97.8)	
**Type of cancer**			
Cancer of the cervix	8 (50)	47 (51.1)	
Cancer of the endometrium	3 (18.8)	19 (20.6)	
Cancer of the ovary	0	15 (16.3)	0.015
Cancer of the vulva	1 (6.3)	11 (12.0)	
Cancer of the vagina	4 (25)	0	
**Cancer staging**			
Stage 1	0	4 (4.3)	
Stage 2	2 (12.5)	14 (15.2)	
Stage 3	5 (31.3)	55 (59.8)	0.024
Stage 4	9 (56.3)	19 (20.7)	
**Type of treatment**			
Surgery	2 (12.5)	17 (18.5)	
Chemotherapy	12 (75)	40 (43.5)	
Radiation	2 (12.5)	29 (31.5)	0.119
Hormonal therapy	0	6 (6.5)	
**Duration of treatment**			
Less than 6 months	10 (62.5)	42 (45.7)	0.059
7–12 months	6 (37.5)	25 (27.2)	
More than 12 months	0	25 (27.2)	

**Table 5.** Socio-demographic, clinical, psychological and social predictors of sexual function.

	*p*-value	OR	95% CI for OR	
			**Lower**	**Upper**
**Age**				
Less than 50 years	0.004	0.142	0.038	0.533
≥50 years		Ref		
**Level of education**				
None		Ref		
Primary	0.999	0.871	0.123	0.671
Secondary	1	0	0.451	0.89
College	1	0.2	0.008	0.141
Degree	1	0.2	0.067	0.201
**Employment status**				
Unemployed		Ref		
Employed	0.002	0.321	0.002	0.641
Self-employed	0	0.095	0.027	0.339
**Marital status**				
Single		Ref		
Married	0.729	0.6	0.033	10.822
Divorced	0.287	0.318	0.039	2.624
Separated	0.209	0.133	0.006	3.081
Widowed	0.999	0.134	0	-
**Lifestyle adaptation activities**				
None		Ref		
Drinking	0.381	0.81	0.41	0.671
Physical activities	0.999	0.002	0.001	0.114
**Type of gynaecological cancer**				
Cancer of the vagina		Ref		
Cancer of the cervix	0.016	7.833	1.469	13.15
Cancer of endometrium	0.018	5.12	1.498	10.45
Cancer of vulva	0.999	0.341	0.095	1.102
Cancer of the ovary	0.089	9.333	0.711	2.57
**Stage of cancer**				
Stage 1	0.058	Ref		
Stage 2	0.999	1.3	0	1.45
Stage 3	0.162	3.316	0.618	17.8
Stage 4	0.008	5.211	1.552	17.495
**Method of cancer treatment**				
Hormonal therapy		Ref		
Surgery	0.999	0.123	0.1	0.561
Chemotherapy	0.999	0.311	0	0.671
Radiation	0.999	0.11	0	0.312
**Duration of treatment**				
Less than 6 months		Ref		
7–12 months	0.998	0	0	0.402
More than 12 months	0.998	0	0	0.251
**Body image**	0.231	0.96	0.899	1.026
**Social support**	0.037	1.102	1.006	1.026

**Table 6.** Independent predictors of sexual function.

Factors	aOR(95%CI)	*p*-value
**Age**		
Less than 50 years	0.05 (0.003,0.16)	0.005
≥50 years	Ref	
**Employment status**		
Unemployed	Ref	
Employed	0.000	0.998
Self employed	0.07 (0.005,1.01)	0.051
**Cancer staging**		
Stage 1	Ref	
Stage 2	0.000	0.998
Stage 3	9.81 (1.34,20.56)	0.035
Stage 4	14.48 (0.94,22.57)	0.055
**Type of cancer**		
Cancer of the vagina	Ref	
Cancer of the cervix	18.47 (0.8,23.511)	0.068
Cancer of endometrium	33.94 (0.53,21.51)	0.098
Cancer of vulva	0.000	0.998
Cancer of the ovary	0.66 (0.013,33.5)	0.836
**Social support**	1.29 (1.05,1.59)	0.015

## References

[1] Mohammed F, Shahin M, Youness E (2018). Survivorship in women undergoing gynecological and breast cancer treatment in upper Egypt: the impact of quality of life improvement educational program. Am Res J Gynaecol.

[2] Sung H, Ferlay J, Siegel RL (2020). Global cancer statistics 2020: GLOBOCAN estimates of incidence and mortality worldwide for 36 cancers in 185 countries. CA Cancer J Clin.

[3] Nady F, El-Sherbiny M, Youness E (2018). Effectiveness of quality of life planned teaching program on women undergoing gynecologic cancer treatment. Am Res J Oncolo.

[4] Globocan (2020). Kenya Source.

[5] Frimer, Turker LB, Shankar V (2019). The association of sexual dysfunction with race in women with gynecologic malignancies. Gynecol Oncol Rep.

[6] Boa R, Grénman S (2018). Psychosexual health in gynecologic cancer. Int J Gynecol Obstet.

[7] Sekse RJT, Hufthammer KO, Vika ME (2017). Sexual activity and functioning in women treated for gynecological cancers. J Clin Nurs.

[8] Hassan H, Bayoumi M, Atwa A (2016). Emotional distress associated with gynecologic and breast cancer in Beni-Suef city. Int J Sci Res.

[9] Hassan H (2020). Early stage cervical cancer: survivorship and fertility preservation. Am Res J Oncol.

[10] Hassan H, Ramadan S, Ali R (2021). Sexual issues among cervical cancer survivors’ women in northern upper Egypt. J Adv Trends Basic Appl Sci.

[11] Hassan H, Mohammed R, Ramadan S (2021). Call for alleviating sexual issues among cervical cancer survivors’ women in northern upper Egypt. J Obstet Gynecol Reproduct Sci.

[12] Qalawa S, Eldeeb A, Hassan H (2015). Young adult women’s intention regarding breast and cervical cancer screening in Beni-Suef. Sci Res J.

[13] Elzeblawy H, Kamal H, Abd El-Salam S (2021). Survivors from cervical cancer: impact of an educational program on self-knowledge and body-image. Public Health Open Acc.

[14] Willows K, Lennox G, Covens A (2016). Fertility-sparing management in cervical cancer: balancing oncologic outcomes with reproductive success. Gynecol Oncol Res Pract.

[15] Hassan H, Masaud H, Mohammed R (2021). Self-knowledge and body image among cervical cancer survivors’ women in northern upper Egypt. Furth Appl Healthc.

[16] Khalil J, Bellefqih S, Sahli N (2015). Impact of cervical cancer on quality of life: beyond the short term (results from a single institution). Gynecol Oncol Res Pract.

[17] Hassan H, Mohammed R, Ramadan S (2021). Impact of an educational program on sexual issues among cervical cancer survivors’ women in northern upper Egypt. J Obst Gynecol Reproduct Sci.

[18] Cleary V, Hegarty J, McCarthy G (2011). Sexuality in Irish women with gynecologic cancer. Oncol Nurs Forum.

[19] Nady F, Said M, Youness E (2017). Impact of tailored educational program of quality of life improvement on women undergoing breast cancer treatment at El-Minia Region, Egypt. Am Res J Gynaecol.

[20] Carr S (2015). International journal of gynecology and obstetrics psychosexual health in gynecological cancer. Int J Gynecol Obstet.

[21] Abd El Salam S, Hassan H, Kamal K (2021). Women’s sexual dysfunction associated with cervical cancer. Appl Sci Comp Math.

[22] Hassan H, Ali R, Abd El Salam S (2021). Impact of an educational program on sexual dysfunction associated with cervical cancer. J Cancer Res Treat.

[23] Shankar A, Prasad N, Roy S (2017). Sexual dysfunction in females after cancer treatment: an unresolved issue. Asian Pacific J Cancer Prev.

[24] Masaud H, Abd Rabo R, Ramadan S (2021). Impact of protocol of nursing intervention on sexual dysfunction among women with cervical cancer. J Nurs Sci Benha Univ.

[25] El Salam A, Ali R, Hassan H (2021). Outcome of an educational program on body image distress associated with cervical cancer. J Adv Trends Basic Appl Sci.

[26] Kamal H, Ali R, Abd El Salam S (2021). Self-knowledge among women with cervical cancer. J Cancer Res Treat.

[27] Ali R, Kamal H, Hassan H (2021). Impact of an educational program on sexual distress associated with cervical cancer. Furt Appl Healthc.

[28] Zagloul M, Naser E, Hassan H (2020). Cervical cancer knowledge, attitude, and practices: educational program management for female workers at Port Said University. Int J Stud Nurs.

[29] Said S, Hassan H, Sarhan A (2018). Effect of an educational intervention on women’s knowledge and attitude regarding cervical cancer. Am J Nurs Res.

[30] Dowson M, Shah NM, Rinko RC (2019). The evaluation and management of female sexual dysfunction. J Fam Pract.

[31] Ali R, Abd El Salam S, Kamal H (2021). Women with cervical cancer: impact of an educational program their knowledge. J Obstet Gynecol Reproduct Sci.

[32] Mansour SE, Mohamed HE (2015). Handling sexuality concerns in women with gynecological cancer: Egyptian nurse’s knowledge and attitudes. J Educ Pract.

[33] Nady F, Said M, Youness E (2018). Effect of nursing intervention program on quality of life improvement for women undergoing gynecological and breast cancer treatment. Assuit Sci Nur J.

[34] Atwa A, Hassan H, Ahmed S (2019). The impact of a hospital-based awareness program on the knowledge of patients about breast cancer and cancer cervix. Int J Stud Nurs.

[35] Mohamed A, Hassan H, Gamel W (2019). Awareness about breast and cervical cancers among nursing students in Beni-Suef University. J Nurs Educ Pract.

[36] Grover S, Hill-Kayser CE, Vachani C (2012). Patient-reported late effects of gynecological cancer treatment. Gynecol Oncol.

[37] Allen KR, Roberto KA (2014). Older women in Appalachia: experiences with gynecological cancer. Gerontologist.

[38] Carter J, Stabile C, Gunn A (2013). The physical consequences of gynecologic cancer surgery and their impact on sexual, emotional, and quality of life issues. J Sex Med.

[39] Ramadan S, Hassan H, Masaud H (2021). Women’s body image distress associated with cervical cancer. J Obstet Gynecol Reproduct Sci.

[40] Masaud H, Hassan H, Mohammed R (2021). Women’s sexual distress associated with cervical cancer. Sumer J Med Healthc.

[41] Ganavadiya R, Shekar BC, Suma S (2018). Effectiveness of two psychological intervention techniques for de-addiction among patients with addiction to tobacco and alcohol – a double-blind, randomized control trial. Indian J Cancer.

[42] Chow KM, Wong CY, Shek LL (2014). Sexual functioning of gynecological cancer patients: a literature review. World J Oncol Res.

[43] Stabile C, Gunn A, Sonoda Y (2015). Emotional and sexual concerns in women undergoing pelvic surgery and associated treatment for gynecologic cancer. Transl Androl Urol.

[44] Huffman LB, Hartenbach EM, Carter J (2016). Maintaining sexual health throughout gynecologic cancer survivorship: a comprehensive review and clinical guide. Gynecol Oncol.

[45] Verschuren JEA, Enzlin P, Dijkstra PU (2010). Chronic disease and sexuality: a generic conceptual framework. J Sex Res.

[46] Zeng YC, Liu X, Loke AY (2012). Addressing sexuality issues of women with gynecological cancer: Chinese nurses’ attitudes and practice. J Adv Nurs.

[47] Ussher JM, Perz J, Gilbert E (2015). Perceived causes and consequences of sexual changes after cancer for women and men: a mixed-method study. BMC Cancer.

[48] Shankar A, Prasad N, Roy S (2017). Sexual dysfunction in females after cancer treatment: an unresolved issue. Asian Pac J Cancer Prev.

[49] Tracy M, McDivitt K, Ryan M (2016). Feasibility of a sexual health clinic within cancer care: a pilot study using qualitative methods. Cancer Nurs.

[50] Lin HC, Lin YC (2018). The study of body image, self-esteem, and sexual satisfaction of college students in Southern Taiwan. Univers J Educ Res.

[51] Li CC, Rew L, Chen L (2015). Factors affecting sexual function: a comparison between women with gynecological or rectal cancer and healthy controls. Nurs Health Sci.

[52] Khoo SB (2009). Impact of cancer on psychosexuality: cultural perspectives of Asian women. Int J Nurs Pract.

[53] Rosen R, Brown C, Heiman J (2000). The female sexual function index (Fsfi): a multidimensional self-report instrument for the assessment of female sexual function. J Sex Marital Ther.

[54] Hopwood P, Fletcher I, Lee A (2001). A body image scale for use with cancer patients. Eur J Cancer.

[55] Rai S (2021). Quality of life and sexual dysfunction in cervical cancer survivors. Nepal J Obstet Gynaecol.

[56] Lara IE, Maree JE (2019). Sexual function in South African women treated for cervical cancer. Int J Africa Nurs Sci.

[57] Pinar G, Kaplan S, Akalin A (2016). Evaluation of sexual dysfunction and affecting factors in Turkish women with gynecological cancer. Sex Disabil.

[58] Liu H, Yu J, Chen Y (2016). Sexual function in cervical cancer patients: psychometric properties and performance of a Chinese version of the Female Sexual Function Index Abstract. Eur J Oncol Nurs.

[59] Pitcher S, Fakie N, Adams T (2020). Sexuality post gynecological cancer treatment: a qualitative study with South African women. BMJ Open.

[60] Leppert W, Gottwald L, Forycka M (2015). Clinical practice recommendations for quality of life assessment in patients with gynecological cancer. Prz Menopauzalny.

[61] Correia RA, Bonfim CV, Santos SL (2020). Sexual dysfunction after cervical cancer treatment. Rev da Esc Enferm.

[62] Zhou W, Yang X, Dai Y (2016). Survey of cervical cancer survivors regarding the quality of life and sexual function. J Cancer Res Ther.

[63] Gray PB, Garcia R (2012). Aging and human sexual behavior: biocultural perspectives – a mini-review. Gerontology.

[64] Kaviani M, Rahnavard T, Azima S (2014). The effect of education on the sexual health of women with hypoactive sexual desire disorder: a randomized controlled trial. Int J Community Based Nurse Midwifery.

[65] Golbasi Z, Erenel AS (2012). The quality of sexual life in women with gynecological cancers. Arch Gynecol Obstet.

[66] Dumas L, Ring A, Butler J (2016). Improving outcomes for older women with gynecological malignancies. Cancer Treat Rev.

[67] Evans REC, Morris M, Sekhon M (2014). Increasing awareness of gynecological cancer symptoms: a GP perspective. Br J Gen Pract.

[68] Bifulco G, De Rosa N, Tornesello ML (2012). Quality of life, lifestyle behavior and employment experience: a comparison between young and midlife survivors of gynecology early-stage cancers. Gynecol Oncol.

[69] Fischer OJ, Marguerie M, Brotto LA (2019). Sexual function, quality of life, and experiences of women with ovarian cancer: a mixed-methods study. Sex Med.

[70] Muliira RS, Salas AS, Brien BO (2017). Quality of life among female cancer survivors in Africa: an integrative literature review. Asia Pac J Oncol Nurs.

[71] Lee Y, Lim MC, Kim SI (2016). Comparison of quality of life and sexuality between cervical cancer survivors and healthy women. Cancer Res Treat.

[72] Izycki D, Woźniak K, Izycka N (2016). Consequences of gynecological cancer in patients and their partners from the sexual and psychological perspective. Prz Menopauzalny.

[73] Pfaendler KS, Wenzel L, Mechanic MB (2015). Cervical cancer survivorship: long-term quality of life and social support. Clin Ther.

[74] Del Pup L, Villa P, Amar ID (2019). Approach to sexual dysfunction in women with cancer. Int J Gynecol Cancer.

[75] Kew FM, Nevin J, Cruickshank DJ (2004). Psychosexual impact of gynecological malignancy. Obstet Gynecol.

[76] Najjar Abdo CH, Mendes de Oliveira W, Moreira E (2005). The impact of psychosocial factors on the risk of erectile dysfunction and inhibition of sexual desire in a sample of the Brazilian population. Sao Paulo Med J.

[77] Donkers H, Smits A, Eleuteri A (2019). Body mass index and sexual function in women with gynecological cancer. Psychooncology.

[78] Ibfelt EH, Kjær SK, Høgdall C (2013). Socioeconomic position and survival after cervical cancer: influence of cancer stage, comorbidity and smoking among Danish women diagnosed between 2005 and 2010. Br J Cancer.

[79] Zimet GD, Powell SS, Farley GK (1990). Psychometric characteristics of the multidimensional scale of perceived social support. J Personality Assessment.

[80] Williams P, Murchie P, Bond C (2019). Patient and primary care delays in the diagnostic pathway of gynaecological cancers:a systematic review of influencing factors. Br J Gen Practice.

